# Open Ring Sign Diagnostic of Multiple Sclerosis in the Emergency Department

**DOI:** 10.5811/westjem.2015.4.26314

**Published:** 2015-06-23

**Authors:** Thomas M. Nappe, Matthew T. Niehaus, Terrence E. Goyke

**Affiliations:** Lehigh Valley Hospital-Muhlenberg, Department of Emergency Medicine, Bethlehem, Pennsylvania

A 26-year-old female presented to the emergency department with a chief complaint of dizziness. Further history revealed that she was experiencing generalized weakness and intractable vomiting for three days, without complaint of abdominal pain or lower gastrointestinal symptoms. Physical examination uncovered mild dehydration with stable vital signs and non-fatigable, horizontal nystagmus consistent with internuclear opthalmoplegia. Computed tomography of her brain was ordered and revealed an “open ring sign” as displayed in the figure.

The “open ring sign,” or open ring enhancement of a lesion on neuroimaging, has been found to be highly specific for demyelinating diseases and can help differentiate them from malignant and infectious neurological disorders, where ring enhancement is more often closed.^1^–^2^ The open ring is typically crescent-shaped and open to the basal ganglia or, as in our patient, the cortex, with the enhanced area resembling acutely inflamed white matter while the unenhanced area resembles more chronic inflammation.^1^ A retrospective case series has found that open ring enhancement has a specificity of 84.4–93.8% for demyelinating conditions, with a likelihood ratio of 5.2 and 17.2 for demyelination over malignancy and infection, respectively.^1^–^2^ In our patient, a demyelinating condition was suspected based on this image, and the diagnosis of multiple sclerosis was ultimately confirmed by the presence of oligoclonal bands on cerebrospinal fluid analysis.

## Figures and Tables

**Figure:**
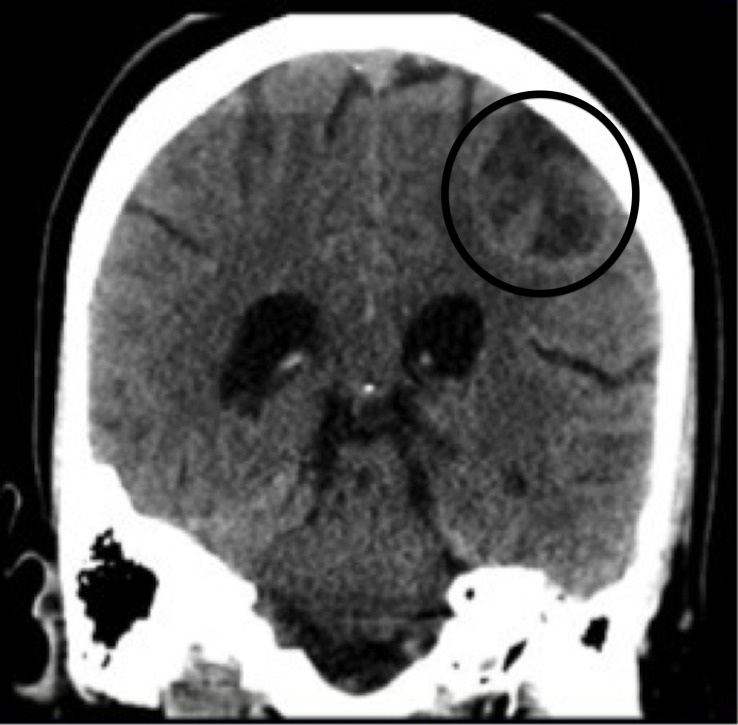
Computed tomography of brain with “open ring sign“ indicated by circle.
